# T2 mapping for the detection of myocardial edema in patients with acute myocarditis

**DOI:** 10.1186/1532-429X-14-S1-P184

**Published:** 2012-02-01

**Authors:** Yoko Mikami, Matthias G Friedrich, Naeem Merchant

**Affiliations:** 1 Stephenson CMR centre, Libin Cardiovascular Institute of Alberta, Calgary, AB, Canada; 2 Diagnostic Imaging and Cardiac Sciences, University of Calgary, Calgary, AB, Canada; 3 Department of Cardiology, Université de Montréal, Montreal, QC, Canada

## Summary

Patients with acute myocarditis and healthy volunteers were studied to assess the ability of T2 mapping to detect myocardial edema. The T2 maps detected global and regional edema in patients. T2 mapping may be an alternative to STIR images for detecting myocardial edema in patients with acute myocarditis.

## Background

In patients with acute myocarditis, T2-weighted cardiovascular magnetic resonance (CMR) can visualize myocardial edema and is used for one of the three recommended diagnostic CMR criteria. There are, however, significant technical problems associated with the short-tau-inversion-recovery (STIR) sequence, limiting its clinical utility. Quantitative T2 mapping technique may overcome such technical limitations and thus improve the diagnostic yield of CMR. Its clinical utility, however, has not been assessed. The purpose of this study is to assess the ability of T2 mapping to detect myocardial edema in patients with acute myocarditis.

## Methods

Ten healthy volunteers and 17 patients with acute myocarditis as diagnosed using clinical and CMR criteria (Lake Louise criteria) and evidence for myocardial edema were studied. CMR studies included STIR images, early and late gadolinium enhanced images and T2-mapping images using a T2-prepared single-shot SSFP acquisition with three T2-prep echo times: 0, 24, and 55 msec. Three short axis T2 mapping images were obtained. Global myocardial T2 values were evaluated on each slice and the mean value of 3 slices were calculated for each subject. Images were also analyzed based on a 16-segment model. T2 values were evaluated in regions of interest in each segment. T2 maps were also visually assessed to determine the location of visually abnormal and remote segments. On the slices where no visually abnormal segments were found, all segments were considered remote.

## Results

The global T2 value in myocarditis patients were significantly higher than those in volunteers (58.5 ± 3.8 vs. 52.4 ± 2.6, p<0.000). Of 402 available segments, 7 segments had to be excluded due to poor image quality. A total of 129 segments in volunteers and 266 in patients were analyzed. Ninety-three segments in patients were visually identified as abnormal on T2 maps. T2 values of visually abnormal segments were significantly higher than segments in volunteers (62.4 ± 5.6 vs. 51.0 ± 4.5, p<0.000) and remote segments (62.4 ± 5.6 vs. 53.5 ± 4.6, p <0.000). In patients, T2 values of remote segments were also significantly higher than those in volunteers (53.5 ± 4.6 vs 51.0 ± 4.5, p <0.000).

## Conclusions

For detecting myocardial edema in patients with acute myocarditis, T2 mapping may be an alternative to T2-weighted STIR imaging.

## Funding

N/A

